# Effect of Polypropylene Fiber Content on the High-Temperature Performance of Steel Slag UHPS

**DOI:** 10.3390/ma19163553

**Published:** 2026-08-21

**Authors:** Jing Wang, Zhiwei Yuan, Yunlong Zhang, Xuesong Qian, Xiaolong Qu

**Affiliations:** 1 School of Transportation Science and Engineering, Jilin Jianzhu University, Changchun 130118, China; 2 Jilin Provincial High Class Highway Construction Bureau, Changchun 130033, China

**Keywords:** UHPS, steel slag, high-temperature resistance, mechanical properties, microstructural characteristics

## Abstract

To solve the problems of explosive spalling and sharp deterioration of mechanical properties of ultra-high performance sprayed concrete (UHPS) under high-temperature conditions during tunnel fires, four groups of specimens with different volume contents (0%, 0.2%, 0.3%, 0.4%) of polypropylene fiber (PPF) were designed based on the optimal mix proportion at room temperature. Multi-gradient high-temperature tests at 20 °C, 200 °C, 400 °C, 600 °C and 800 °C were conducted to explore the effects of PPF on the high-temperature damage evolution and spalling resistance of UHPS. The test results show that no obvious spalling occurs in specimens exposed to temperatures of 400 °C and below. Surface peeling appears in the group without PPF addition at 400 °C, and severe explosive spalling happens in the 0% PPF group at 600 °C to 800 °C, while all PPF-incorporated groups maintain structural integrity. The mass loss rate increases with the rise in temperature and PPF content, reaching 15% in the 0.4% PPF group at 800 °C. In terms of mechanical properties, compressive strength, splitting tensile strength, and flexural strength all rise first and then decline with increasing temperature, and the 0.3% PPF group reaches peak values at 400 °C (compressive strength: 135.95 MPa, splitting tensile strength: 23.24 MPa, flexural strength: 27.42 MPa). Flexural toughness decreases continuously as temperature rises, and the 0.2% PPF group exhibits the best toughness retention. This study clarifies the high-temperature modification effect of PPF on UHPS and its optimal content range, providing important theoretical support and an experimental basis for the fire safety protection design of tunnel lining concrete.

## 1. Introduction

With the accelerated advancement of global underground infrastructure, the construction scale of mountain tunnels, urban subways, and deep-buried tunnels continues to expand. As the primary structural material for the New Austrian Tunneling Method (NATM), shotcrete has become a cornerstone of ground support systems, favored for its operational convenience, robust rock-mass adaptability, and rapid structural support capabilities [1]. Conventional shotcrete is widely utilized; however, its inherent limitations become pronounced under challenging service environments. Specifically, it exhibits high rebound rates, leading to substantial material wastage and diminished construction efficiency. Furthermore, its relatively high matrix porosity and loose interfacial transition zone (ITZ) result in inadequate early-age strength, coupled with suboptimal impermeability and crack resistance [2]. Notably, under extreme conditions such as tunnel fires, conventional shotcrete is susceptible to severe bursting and surface degradation, causing a precipitous loss in load-bearing capacity. Such degradation may culminate in the failure of supporting structures, thereby compromising the long-term operational safety and post-disaster recovery of underground facilities [3].

To overcome these performance bottlenecks, ultra-high performance shotcrete (UHPS) has emerged as a focal point in tunnel engineering research [4]. UHPS is formulated through the optimization of aggregate gradations and the utilization of low water–binder ratios combined with high-performance water reducers. Characterized by exceptional compressive and flexural strength, superior matrix densification, and enhanced durability, UHPS effectively meets the stringent requirements for rapid support and long-term serviceability under complex geological conditions [5]. Currently, existing investigations into UHPS predominantly emphasize fresh workability, construction techniques, and mechanical properties at normal temperature. However, comprehensive research regarding the performance evolution and modification mechanisms of UHPS under elevated temperature environments—particularly the extreme thermal transients associated with tunnel fire—remains limited.

Tunnel fires represent one of the most hazardous threats in underground engineering. Due to the semi-enclosed nature of tunnels, fires often result in severe heat accumulation, with temperatures potentially exceeding 1000 within a brief duration, far surpassing the intensity of typical building fire scenarios [6]. Under such thermal loads, concrete undergoes the dehydration and decomposition of cement hydration products. Thermal mismatch between aggregates and the cement matrix initiates crack propagation, while the rapid vaporization of pore water within the dense matrix structure generates excessive steam pressure, leading to explosive bursting [7]. For UHPS, which features an ultra-dense matrix, this extremely low permeability further exacerbates the risk of high-temperature bursting, representing a critical challenge for its large-scale application in fire-safety-critical tunnel projects.

PPF, an organic synthetic material, offers cost-effectiveness, favorable dispersibility, and chemical stability, making it a common choice for the fire-resistance modification of cement-based materials [8]. With a melting point of approximately 160 °C, PPF melts during the early stages of a fire, generating interconnected pore channels that facilitate the release of internal steam pressure, thereby effectively restraining the bursting of concrete [9]. Additionally, PPF exerts a bridging effect at normal temperature, which refines the pore structure, impedes crack propagation, and enhances the flexural toughness and crack resistance of the material [10]. While the benefits of PPF in ordinary concrete and UHPS are well-documented, their influence on the high-temperature behavior of UHPS warrants further investigation. In particular, the spalling resistance, residual mechanical properties, and underlying modification mechanisms of PPF under realistic tunnel fire temperature profiles remain to be thoroughly elucidated.

Concurrently, the resource-efficient utilization of steel slag—a primary byproduct of the iron and steel industry—represents a significant step toward the low-carbon advancement of cement-based materials. Substituting natural aggregates with steel slag fine aggregate (SSFA) in UHPS not only mitigates the scarcity of natural resources and reduces the carbon footprint of construction but also enhances the compactness and mechanical properties of the matrix. Furthermore, the thermal expansion coefficient of steel slag aggregate demonstrates better compatibility with cement hydration products, which may alleviate the thermal deformation mismatch between the aggregate and the matrix at high temperatures, thereby inhibiting the initiation and development of thermal stress-induced cracks [11,12,13,14].

Previous studies have demonstrated that steel slag aggregate and steel fibers can effectively improve the mechanical performance of UHPS. However, most existing studies have focused on the effects of steel slag aggregate or fiber reinforcement individually, while the influence of polypropylene fibers incorporated into steel slag aggregate and steel fiber-reinforced UHPS under elevated temperatures remains insufficiently explored. In particular, the role of polypropylene fiber dosage in regulating thermal damage evolution, mechanical degradation, and microstructural changes after high-temperature exposure has not been fully clarified.

Therefore, this study investigates the influence of hybrid fibers combined with SSFA on the high-temperature performance of UHPS. The effects of varying PPF dosages on the fresh workability and mechanical properties of UHPS were evaluated. Furthermore, the role of PPF in mitigating high-temperature damage, improving residual mechanical performance, and regulating microstructural evolution was systematically investigated. This study aims to elucidate the modification mechanism of PPF on the thermal performance of UHPS and provide theoretical insights and technical guidance for the application of UHPS in fire-resistant tunnel support systems.

## 2. Materials and Methods

### 2.1. Raw Materials and Mix Proportion

#### 2.1.1. Raw Materials

The raw materials used in this test include cementitious materials, fine aggregates, water reducers, and fibers. The cementitious materials consist of P.O 52.5 cement, metakaolin, silica fume, and fly ash. The fine aggregates are manufactured sand (MS) and steel slag fine aggregate (SSFA). The SSFA used in this study had been naturally aged outdoors for more than one year before use and was subsequently subjected to grinding treatment. This pretreatment was intended to reduce the potential volume instability associated with reactive components in steel slag and to improve the stability of the aggregate before its incorporation into UHPS [15]. The chemical compositions and physical properties of cementitious materials and aggregates are shown in Table 1, Table 2 and Table 3. The adopted superplasticizer is a polycarboxylic acid product purchased from Sichuan Dongrun Baisheng New Materials Co., Ltd. (Chengdu, China).

**Figure 1:**
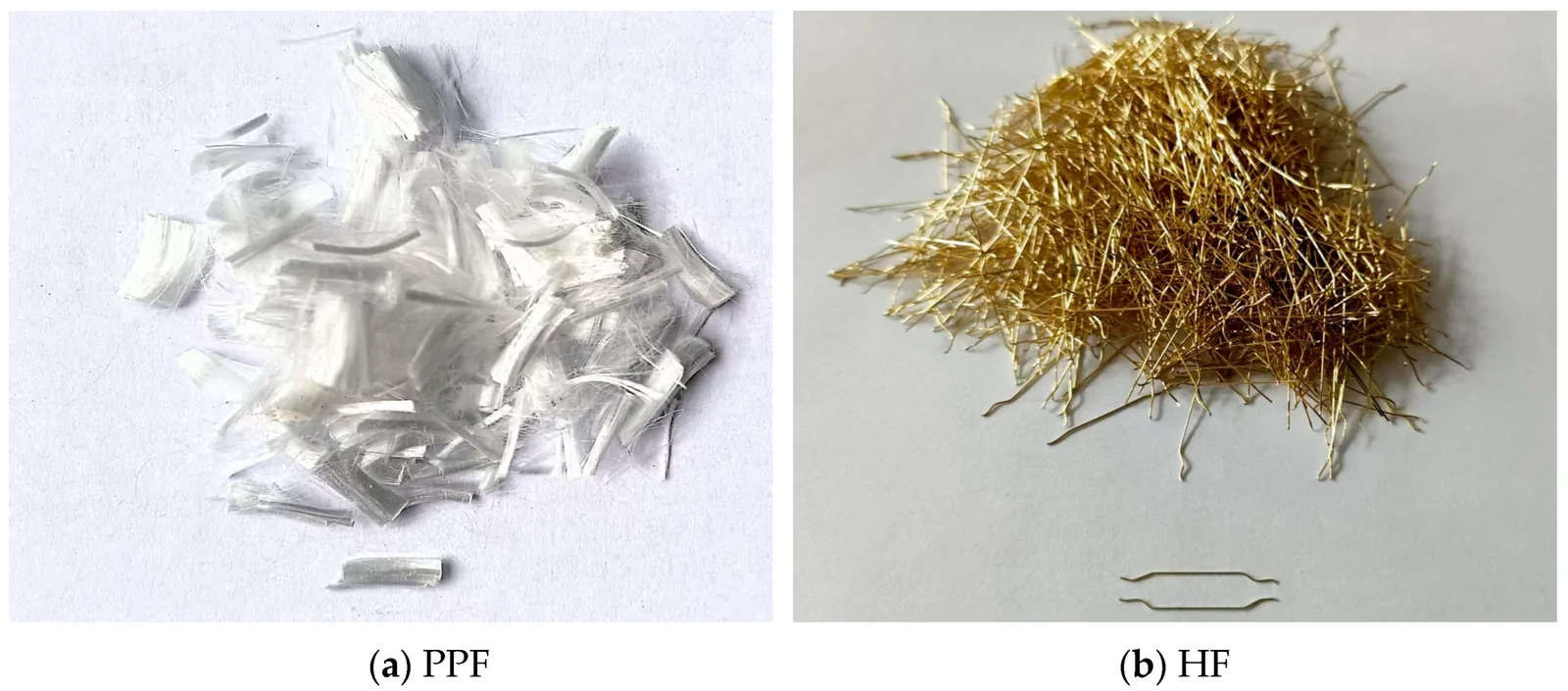
Fibers.

The polypropylene fiber (PPF) was purchased from Beijing Zhongfang Fiber Construction Technology Co., Ltd. (Beijing, China) (Kaitai modified polypropylene ECC fiber), while the copper-plated hooked-end steel fiber (HF) was manufactured by Liaocheng Hongshengyuan Metal Products Co., Ltd. (Liaocheng, China). The physical properties of the fibers are listed in Table 4, and their appearances are shown in Figure 1.

#### 2.1.2. Mix Proportion Design

The control mix design utilizes a water–binder ratio of 0.2, a sand–binder ratio of 0.9, an SSFA content of 75%, and 2% hooked-end steel fiber (HF). Based on this baseline, four experimental series were designed with PPF volume fractions of 0%, 0.2%, 0.3%, and 0.4%. The five target temperature levels evaluated were 20 °C, 200 °C, 400 °C, 600 °C, and 800 °C. Detailed mixture proportions are provided in Table 5.

### 2.2. Specimen Preparation and Test Methods

#### 2.2.1. Specimen Preparation

**Figure 2:**
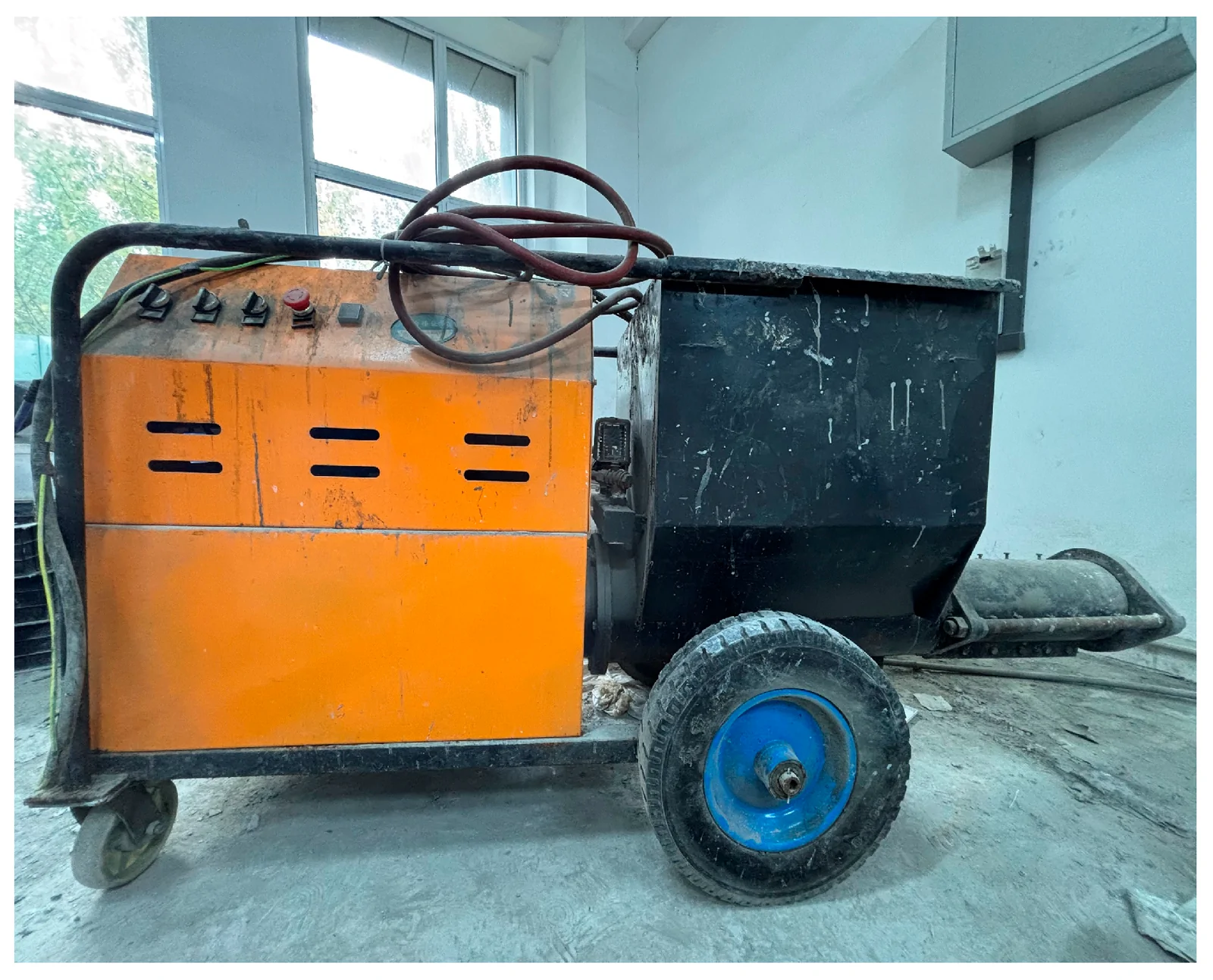
Spraying equipment.

Specimens were prepared in accordance with the Chinese standard JGJ/T 372-2016 [16]. Initially, all cementitious components were dry-mixed for 2 min. PPF was subsequently added, and mixing continued to ensure uniform dispersion. Water and the polycarboxylate-based high-performance water reducer were premixed and added to the solids, followed by a 2 min mixing cycle. Steel fibers were then added via a sieve to prevent clumping. After an additional 4 min of mixing, the UHPS slurry was ready for application. The spraying process was conducted using a wet-mix shotcrete machine by Hubei Locomotive Huanfa Machinery Equipment Co., Ltd. (Wuhan, China) with a rated voltage of 380 V, a working pressure of 0.8 MPa, and a main motor power of 7.5 kW. The maximum aggregate size allowed by the equipment was 1–8 mm, and the inner diameter of the conveying pipe was 50 mm. Spraying was performed at a nozzle-to-substrate distance of 0.6–1.0 m and an orientation angle of approximately 80 degrees. After spraying was completed, the specimens were subjected to standard curing for 24 h, followed by demolding and outdoor natural curing until the specified testing age (3 d, 7 d, 14 d, and 28 d). During the outdoor curing period, the ambient temperature ranged from 16 to 28 °C, with an average relative humidity of approximately 65%. Large plate samples were cut into the specified test geometries using an automatic pave saw manufactured by Matest S.p.A. (Treviolo, Italy). The spraying setup is illustrated in Figure 2.

#### 2.2.2. High-Temperature Test

**Figure 3:**
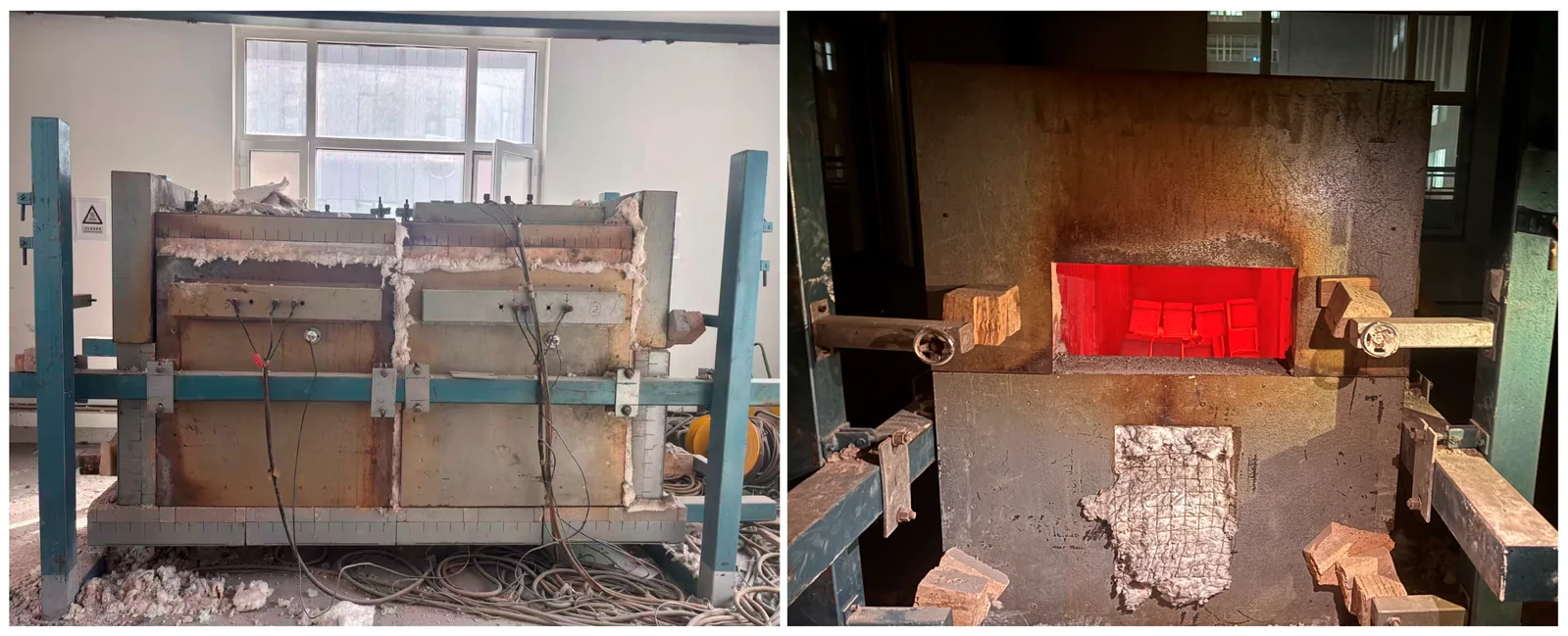
High temperature testing equipment.

For each temperature increment, three replicate specimens were tested to ensure data robustness. After curing, specimens were air-dried and surface-cleaned prior to thermal exposure. The selected target temperatures—20 °C, 200 °C, 400 °C, 600 °C, and 800 °C—simulate realistic tunnel fire temperature gradients. The high-temperature apparatus is shown in Figure 3. A programmed thermal cycle was implemented: specimens were heated from normal temperature to the target temperature at a rate of 10 °C/min, maintained at the target temperature for 2 h, and then allowed to cool naturally to normal temperature within the furnace prior to mechanical testing.

#### 2.2.3. Mechanical Properties

**Figure 4:**
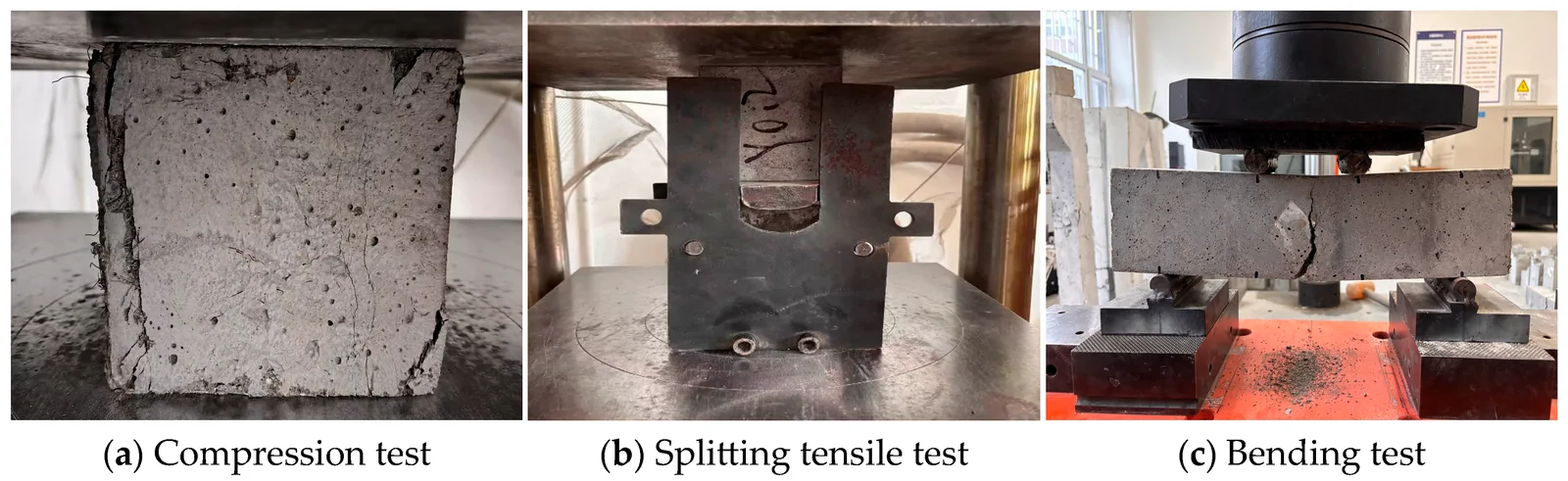
Test Process.

As illustrated in Figure 4, mechanical tests were performed following GB/T 50081-2019 [17]. Compressive and splitting tensile tests utilized 100 mm × 100 mm × 100 mm cubic specimens, while flexural tests utilized 100 mm × 100 mm × 400 mm specimens. Three specimens were prepared for each test condition. Loading rates were set to 1.0 MPa/s for compression, 0.1 MPa/s for splitting tension, and 0.1 mm/min for the four-point bending test. The loading equipment for this experiment mainly includes two types. One is the YAD-3000 microcomputer-controlled electro-hydraulic servo pressure testing machine produced by Jilin Guanteng Automation Technology Co., Ltd. (Changchun, China). The maximum test force of this equipment is 3000 kN, the relative error of the indication is ≤±1%, and the displacement resolution can reach 0.001 mm. The second is the JAW-500P self-balancing structural mechanics innovative loading testing system developed by Hangzhou Bangwei Electromechanical Control Engineering Co., Ltd. (Hangzhou, China). The system has a rated maximum vertical and horizontal loading force of 500 kN, an indication accuracy of ≤± 1%, and a displacement measurement resolution of 0.01 mm.

## 3. Results and Discussion

### 3.1. Appearance Changes

**Figure 5:**
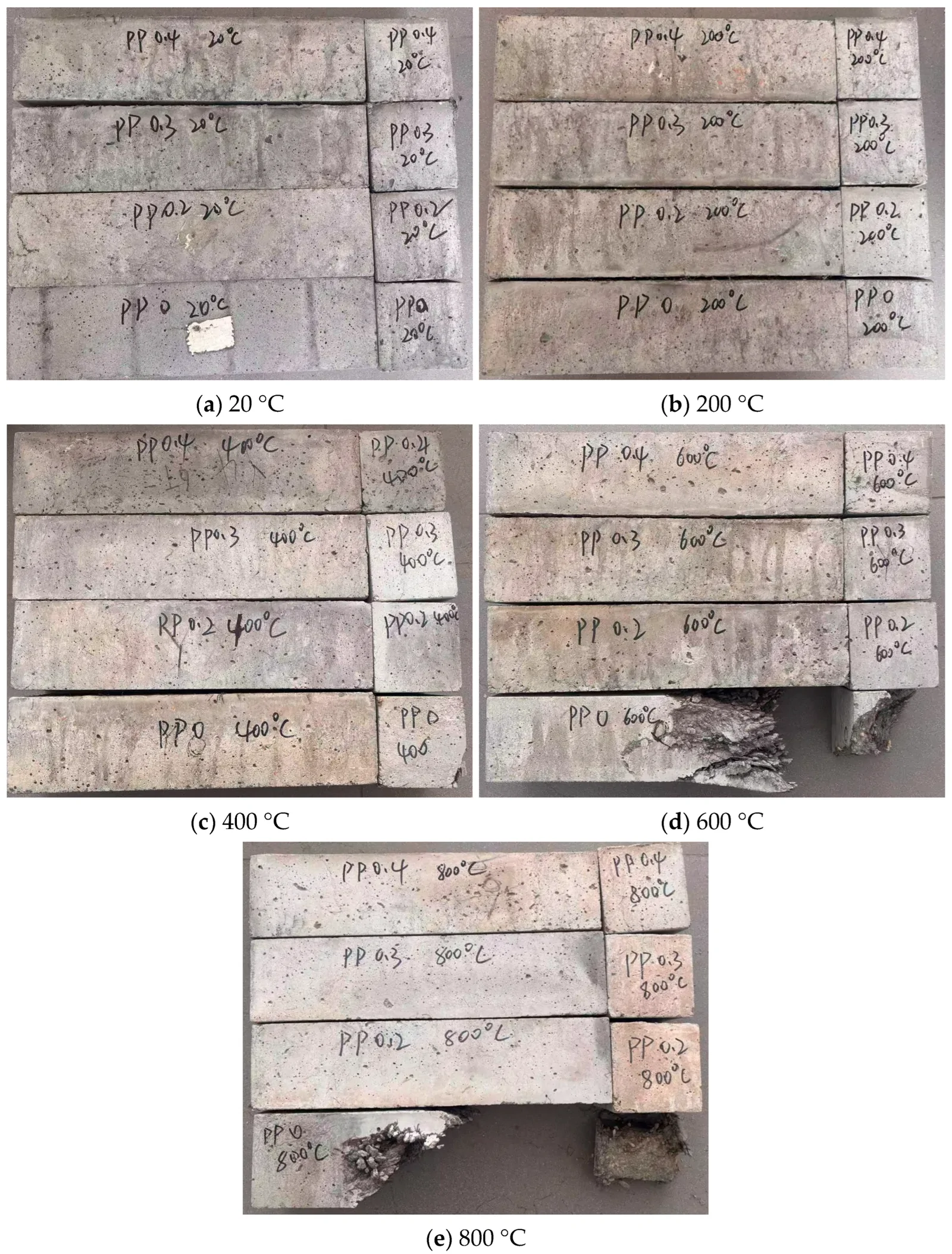
Surface changes of high-temperature specimens.

Figure 5 displays the visual evolution of the specimens under the five investigated temperatures. Color variations reflect internal microscopic chemical changes [18]. At normal temperature (20 °C), specimens exhibit a uniform gray appearance. At 200 °C, the surface shifts to a blue–gray hue, indicating the densification of C-S-H gel as internal hydration continues. Upon reaching 400 °C, the color fades to off-white, reflecting the loss of adsorbed and chemically bound water. By 600 °C, the surface appears off-white with yellowish tints, signaling the dehydration of cementitious hydration products and the brittleness of steel fibers. At 800 °C, pale yellowish-white hues and black-spotted regions emerge, corresponding to high-temperature carbonization, steel fiber oxidation, and further decomposition of the matrix constituents. At this stage, the steel fibers exhibit an almost complete loss of toughness.

Figure 5 visually presents the appearance evolution characteristics of specimens with four different PPF contents under five temperature gradients of 20 °C (normal temperature), 200 °C, 400 °C, 600 °C, and 800 °C. Combined with the observations during the test, no spalling or bursting occurred in any specimens regardless of PPF incorporation within the temperature range of 400 °C and below. This is because within this temperature range, water evaporation dominates inside UHPS with a relatively gentle evaporation rate, resulting in low vapor pressure that does not exceed the bearing limit of the UHPS matrix. The matrix can release pressure through its own pores.

At 400 °C, as shown in Figure 5c, PPF-reinforced specimens remained intact, whereas specimens without PPF experienced surface spalling after 2 h of thermal soaking. The mass evaporation of chemically bound water at this temperature generates significant internal vapor pressure. In PPF-modified specimens, the pre-melted fibers provided critical pathways for moisture egress, preventing pressure buildup [19]. Conversely, the control group lacked these pressure-relief channels, leading to surface spalling once internal pressure exceeded the material’s tensile strength.

When the temperature further rises to 600 °C and 800 °C, as shown in Figure 5d,e, obvious explosive spalling occurs in all specimens without PPF incorporation (0% PPF), and the degree of spalling at 800 °C is significantly more severe than that at 600 °C. Specific experimental observations show that after being kept at a constant temperature of 600 °C for half an hour, cracks and bursting began to occur in the 0% PPF specimens, and the bursting phenomenon ceased after 1.5 h of constant temperature treatment. At 800 °C, cracking and bursting sounds were heard as soon as the temperature rose to 600 °C, and all 0% PPF specimens completely burst after the test ended. With the increase in temperature, a large number of hydration products of cement paste (such as C-S-H gel and calcium hydroxide) undergo severe dehydration and decomposition, generating obvious thermal stress. Meanwhile, the residual internal moisture evaporates rapidly and forms tremendous vapor pressure. Specimens without PPF incorporation lack effective pressure relief channels, so the superposition of stress and pressure leads to cracking and bursting of the matrix. Moreover, high temperature at 800 °C causes more thorough damage to the internal structure of UHPS and produces greater thermal stress and vapor pressure, resulting in more violent bursting behavior. For all specimens mixed with PPF, the pre-formed pore channels continuously exerted a pressure relief effect, which effectively alleviated the accumulation of steam pressure. Meanwhile, the residual pores formed by melted PPF could disperse thermal stress. Therefore, these specimens maintained intact structures without obvious spalling and bursting phenomena [20].

### 3.2. Mass Loss

**Figure 6:**
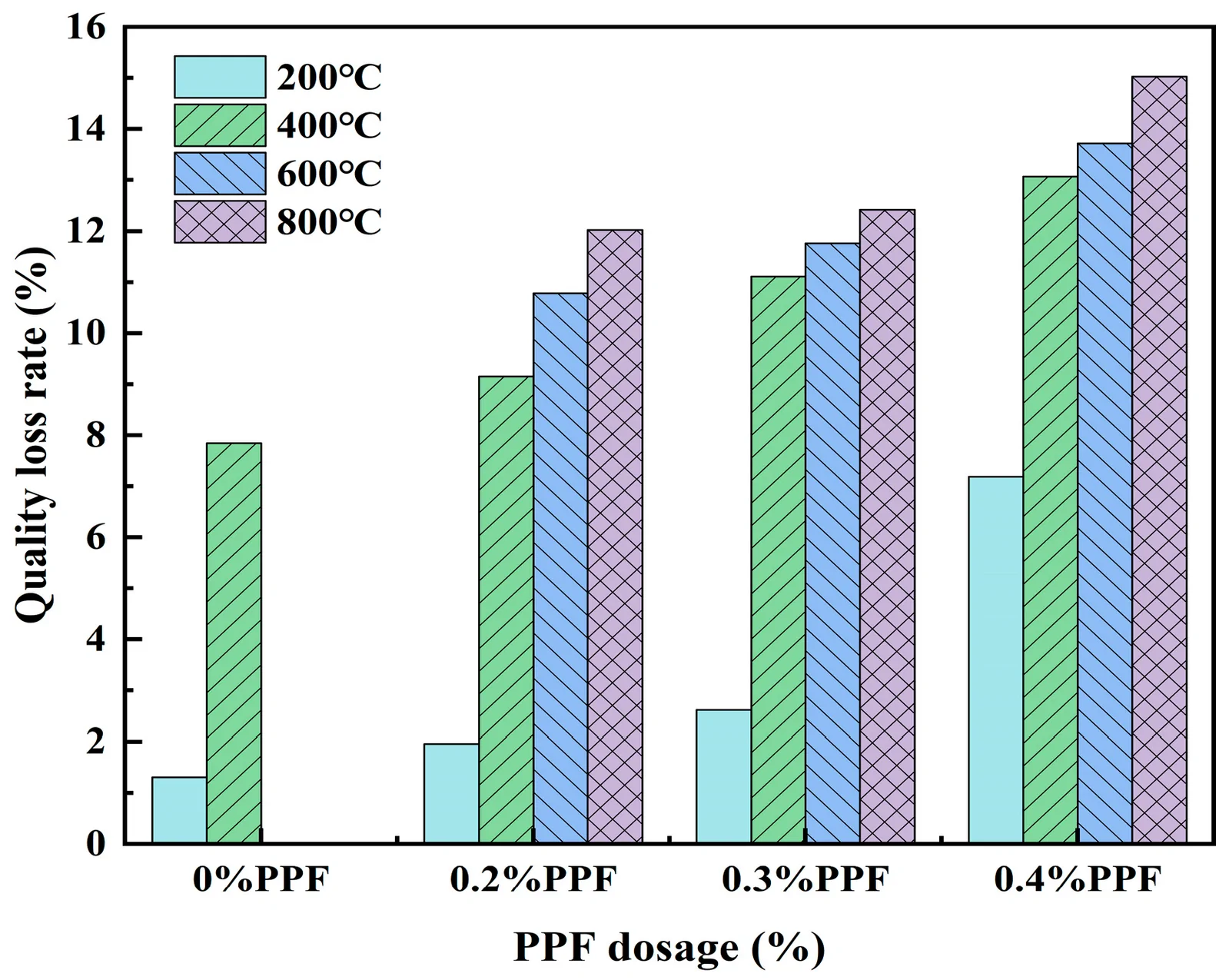
Quality loss after high temperature.

Under high temperature, the mass loss of concrete results from the combined effects of multiple factors such as water evaporation, melting and escape of PPF, and decomposition of hydration products [21]. The degree of mass loss directly reflects the damage evolution process inside the material, and the relevant test data are shown in Figure 6. It should be noted that specimens without PPF incorporation suffered severe spalling at 600 °C, resulting in the destruction of structural integrity, so subsequent quantitative analysis of mass loss cannot be carried out.

The mass loss rate increased significantly with temperature. At the same temperature, the mass loss rate also increased with PPF dosage, with the 0.4% PPF group exhibiting the highest value at 800 °C [22]. The causes are twofold: First, higher temperatures drive the loss of adsorbed and chemically bound water. PPF-modified specimens exhibit slightly higher mass loss in the 200–400 °C range because the interconnected pores facilitate more efficient moisture transport. At 600–800 °C, the decomposition of Ca(OH)_2_ and C-S-H gel, together with minor surface peeling, becomes the primary cause of the sharp increase in mass loss [23].

Secondly, PPF dosage significantly influences mass loss [24]. Higher fiber dosages produce a greater volume of evacuated material and larger porous networks. While these networks successfully mitigate bursting, they reduce matrix compactness, leading to slight surface degradation at high temperatures. Notably, despite the 15% mass loss observed in the 0.4% PPF group at 800 °C, structural integrity was preserved, demonstrating the effectiveness of PPF as a high-temperature modification agent [25,26].

The coefficients of variation (COV) of the mass loss ratio ranged from 0.004 to 1.732. The highest COV was observed in the 200 °C group, which is mainly attributed to the fact that the mean mass loss ratio at this temperature was very close to zero, leading to a significant amplification of the COV value.

### 3.3. High-Temperature Mechanical Properties

#### 3.3.1. Compressive Strength

**Figure 7:**
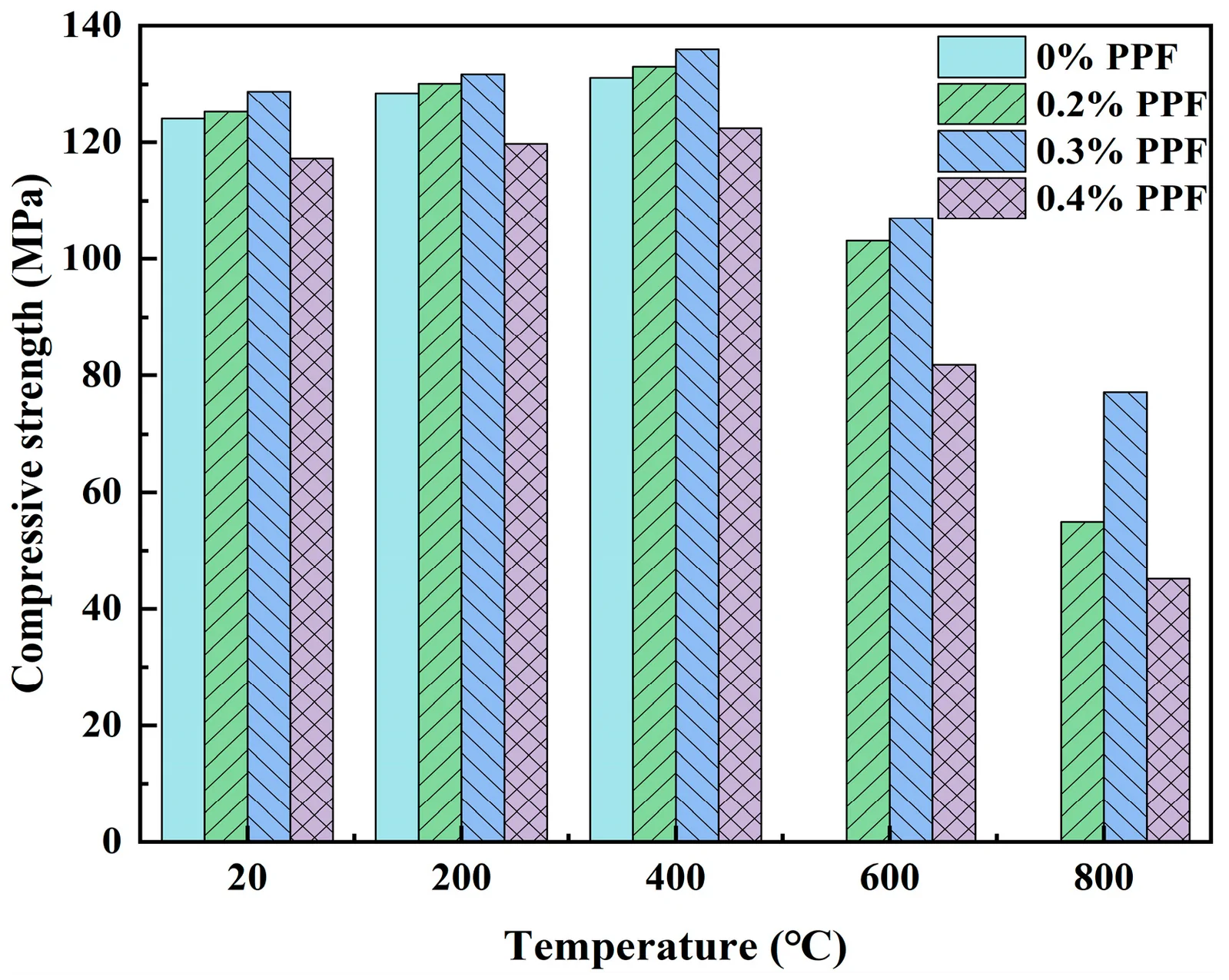
High-temperature compressive strength.

Compressive strength is a primary indicator of load-bearing safety. This section evaluates the influence of PPF content (0–0.4%) across temperature gradients (20–800 °C). Data are presented in Figure 7. Specimens were tested at normal temperature after cooling from the target thermal exposure. The COV of the compressive strength results ranged from 0.006 to 0.143, with only five groups exceeding 0.1. Overall, the compressive strength results exhibited acceptable consistency, supporting the reliability of the experimental data.

PPF content significantly influences compressive strength. At normal temperature, strength initially increases and then decreases with fiber loading, peaking at 128.73 MPa for the 0.3% PPF group. Beyond 0.3%, the compressive strength decreased to 117.2 MPa at 0.4% PPF, which was attributed to fiber agglomeration and the formation of internal defects [27]. At elevated temperatures, the influence of fiber dosage remains consistent with the normal-temperature trend, with the 0.3% PPF group consistently providing optimal performance. Overall, compressive strength initially increased with temperature and reached a peak of 135.95 MPa at 400 °C for the 0.3% PPF group. It subsequently decreased as the temperature approached 800 °C [28,29]. Between 20 °C and 400 °C, post-hydration processes and matrix densification overcome the minor porosity introduced by fiber melting [30]. Beyond 600 °C, the massive decomposition of C-S-H and Ca(OH)_2_, thermal stress, and steel fiber embrittlement dominate, causing substantial strength degradation [31].

#### 3.3.2. Splitting Tensile Strength

Splitting tensile strength reflects the resilience of UHPS against tensile failure, a critical parameter for tunnel safety [32]. The synergy between steel fibers and PPF is essential for maintaining this property [9]. Similar to compressive strength, the 0.3% PPF group demonstrates optimal tensile strength, while 0.4% fiber loading yields suboptimal results due to agglomeration-induced flaws [33].

**Figure 8:**
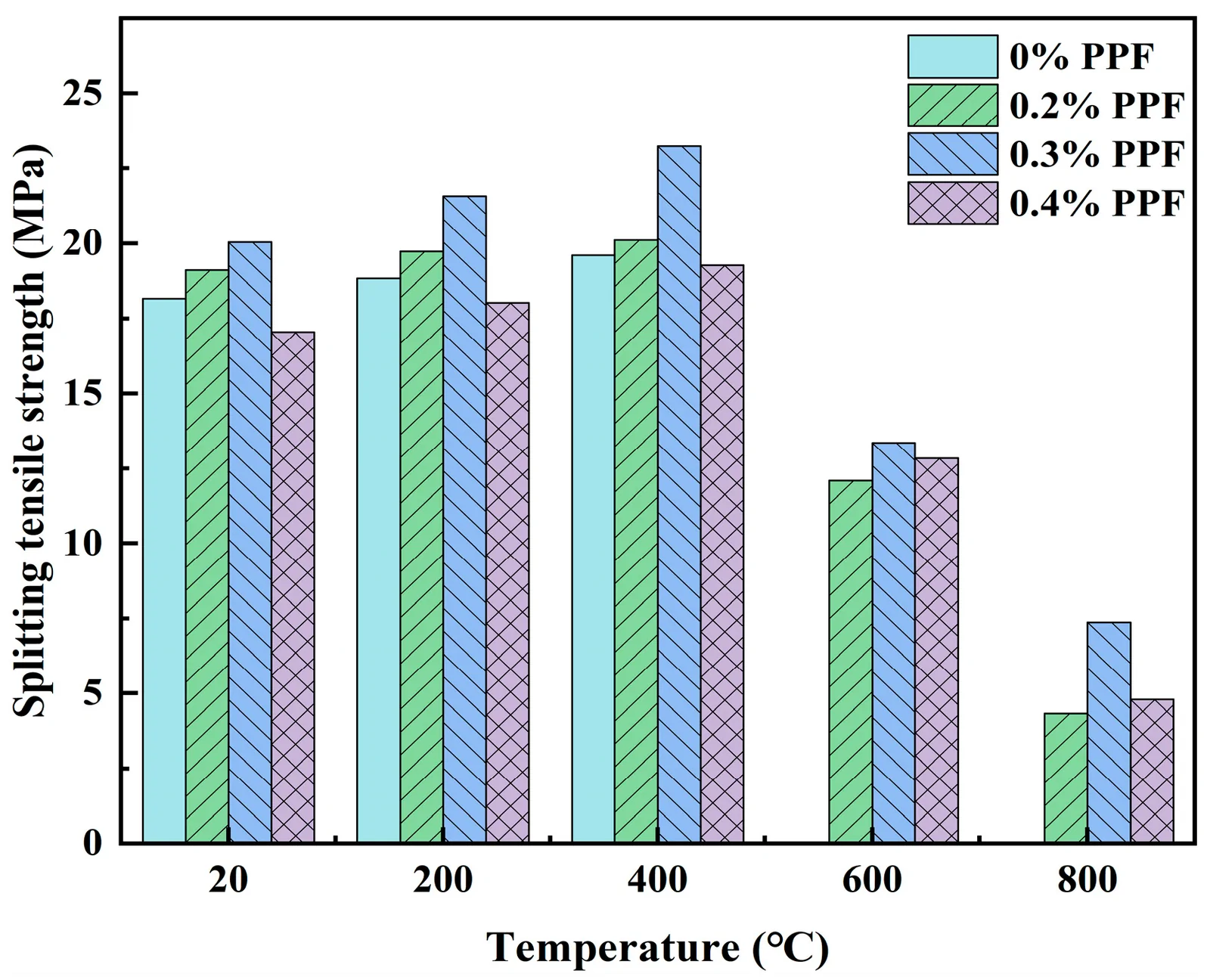
High-temperature splitting tensile strength.

As shown in Figure 8, from the perspective of the influence of temperature gradient, the splitting tensile strength rises from 20 °C to 400 °C with the increase in temperature and shows a continuous decreasing trend after 600 °C. Combined with specific data analysis, the 0.3% PPF group reaches the maximum value of 23.24 MPa in the entire test at 400 °C. This can be attributed to the accelerated hydration reactions within the temperature range of 20–400 °C, which promote the formation of additional C-S-H gel and improve the interfacial transition zone between the cementitious matrix and aggregates. Consequently, the interfacial bonding strength is enhanced [34]. Meanwhile, fewer pore channels are formed by the melting and escape of PPF at this stage, which causes no obvious damage to the tensile bearing structure. In addition, HF still maintains excellent toughness, and its bridging effect can effectively restrain the propagation of microcracks. Above 600 °C, severe dehydration and decomposition of the hydration products occur within UHPS, while phase transformations induce volume shrinkage and substantial thermal stress. Meanwhile, the rapid evaporation of residual internal moisture generates steam pressure. The combined action of the two factors gives rise to a large number of microcracks inside the matrix and accelerates their rapid propagation [35]. Furthermore, with the increase in temperature, the pore channels formed by melted PPF gradually expand and connect with each other, destroying the continuity of the matrix. More importantly, the toughness of HF deteriorates gradually and shows an embrittlement trend as temperature rises, resulting in a sharp decline in splitting tensile strength. At 800 °C, the matrix structure is completely damaged and the interfacial transition zone fully fails. Moreover, the toughness of HF is almost completely lost and the bridging effect ceases to function entirely. Consequently, the splitting tensile strength of the 0.3% PPF group drops to 7.37 MPa, which is merely 31.7% of the maximum value measured at 400 °C.

The variability of the splitting tensile strength results was further evaluated by considering both the COV and the corresponding variance. The COV values ranged from 0.001 to 0.188, while the calculated variances showed a consistent trend with the COV distribution, confirming the reliability of the statistical assessment. Most test groups exhibited very low variance and COV values below 0.10, indicating that the experimental results were tightly clustered around the mean and demonstrated good repeatability. The variation in the COV among the test groups may be attributed to the inherent heterogeneity of UHPS, including variations in fiber dispersion and orientation, aggregate distribution, internal defects, and specimen preparation. For specimens exposed to elevated temperatures, differences in thermal damage, microcrack development, and temperature distribution may further contribute to the dispersion of the measured properties. In addition, testing and measurement procedures may introduce minor variability among replicate specimens.

#### 3.3.3. Flexural Strength

**Figure 9:**
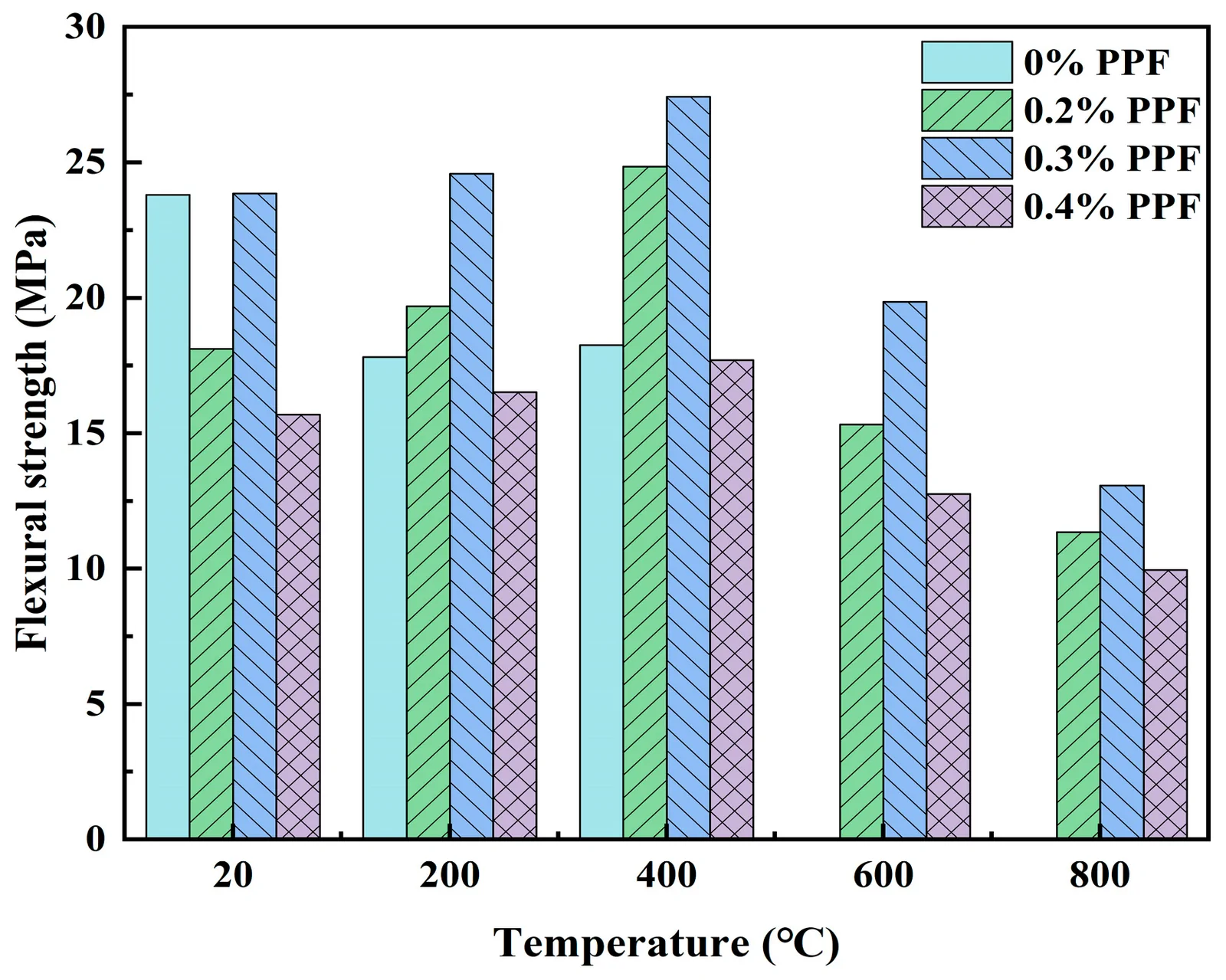
High-temperature flexural strength.

Flexural strength is a key index reflecting the ability of UHPS to resist bending damage at high temperatures. Its damage and failure are primarily associated with the initiation, propagation, and coalescence of internal microcracks under bending load. These processes are also influenced by interfacial bonding and fiber-bridging effects. The evolution law of flexural strength under different temperatures and PPF dosages is shown in Figure 9. The test results show that there are differences in the variation rules among different temperature ranges. In the high-temperature range of 200 °C to 800 °C, the flexural strength of each group first increases and then decreases with the rise in PPF content, reaching the maximum value at the corresponding temperature when the PPF content is 0.3%. This rule is completely consistent with that of compressive strength. This is because an appropriate dosage of PPF (0.3%) can release vapor pressure through pore channels formed during melting, inhibit the development of high-temperature microcracks, and meanwhile generate a synergistic bridging effect with HF to hinder crack propagation and enhance the bending deformation resistance of the matrix [36]. In contrast, excessive PPF (0.4%) tends to agglomerate and form internal defects, damaging the integrity of the matrix. Under bending load, these defects easily become weak paths for crack growth, which in turn leads to a reduction in flexural strength. At room temperature (20 °C), the flexural strength of the four groups of specimens shows a trend of decreasing first, then increasing, and finally decreasing. The specific values are 23.81 MPa for the 0% PPF group, 18.11 MPa for the 0.2% PPF group, 23.86 MPa for the 0.3% PPF group, and 15.69 MPa for the 0.4% PPF group. This phenomenon stems from the fact that the influence of fibers on the flexural properties at room temperature depends on their dispersibility and interfacial bonding. When the content of PPF is 0.2%, it is difficult for fibers to disperse evenly in the concrete matrix, and they tend to be sparsely distributed in local areas or form slight agglomerations. Effective bridging cannot be formed in sparse areas to restrain crack propagation, while the slightly agglomerated areas become internal weak points where stress is prone to concentrate under flexural loads, which in turn reduces the overall flexural strength. Fibers in the 0.3% PPF group disperse uniformly and can effectively inhibit the propagation of flexural cracks. Fiber agglomeration in the 0.4% PPF group leads to defect formation and lowers the flexural bearing capacity.

Flexural strength peaks at 400 °C for all PPF-modified groups [37], with that of the 0.3% PPF group reaching 27.42 MPa. The positive thermal effect in this range is attributed to secondary hydration and densification [38]. Conversely, after 600 °C, the severe decomposition of the matrix, the loss of ITZ integrity, and steel fiber embrittlement cause a precipitous decline in flexural capacity. The control group without PPF exhibits inferior performance at 200 °C and 400 °C due to accumulated steam pressure-induced ITZ damage. These results confirm that the combined use of HF and PPF effectively improves the residual flexural strength of UHPS exposed to 800 °C [39]. The COV of the flexural strength results ranged from 0.00937 to 0.06562, corresponding to 0.94–6.56%. Most of the test groups exhibited COV below 0.050, indicating low dispersion among the specimens and good repeatability of the flexural strength measurements.

#### 3.3.4. Flexural Toughness

**Figure 10:**
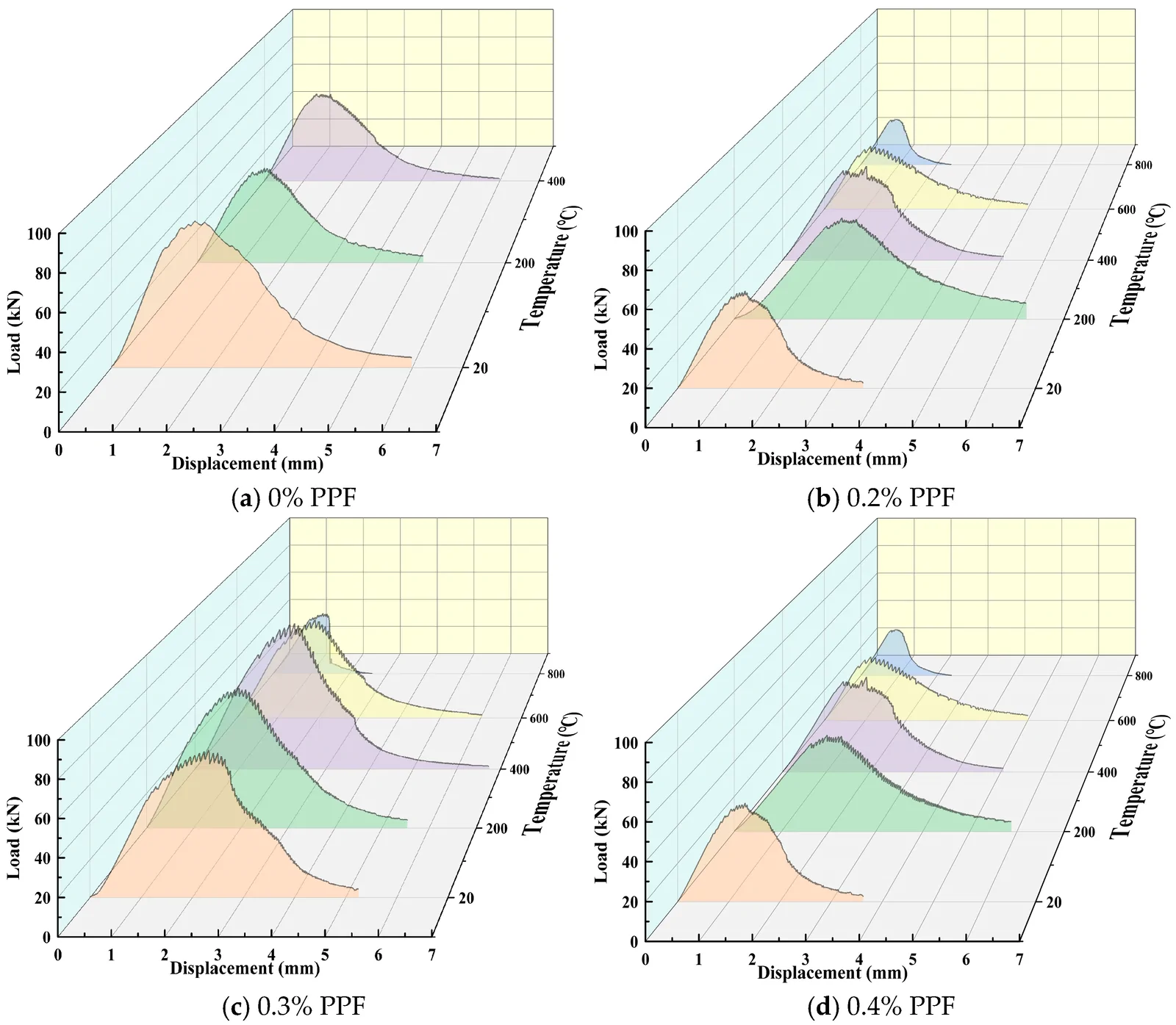
High-temperature load–displacement curve.

As a core index to measure the deformation resistance and energy dissipation capacity of concrete under flexural loading, flexural toughness directly reflects the safety of UHPS in tunnel lining structures against surrounding rock flexural deformation and brittle fracture after high temperature exposure and is of great significance for evaluating the repair value and bearing reliability of structures after fires [40]. This section synchronously tests the initial cracking strength, peak strength, and toughness indicators during the bending process. The test data are shown in Table 6 and Figure 10. In Table 6, Ω_δ_ represents the area under the curve up to the first-crack deflection, corresponding to the energy absorbed before initial cracking, while Ω_3δ_ denotes the cumulative area under the curve up to three times the first-crack deflection. The flexural toughness index I_5_ is defined as the ratio of Ω_3δ_ to Ω_δ_ and is used to characterize the post-cracking load-carrying and energy-absorption capacity of the specimens [41].

From the perspective of temperature gradient influence, under the same PPF content condition, the initial cracking strength and peak strength show completely consistent variation rules. As the test temperature rises from 20 °C to 800 °C, both strengths generally increase first and then decrease, reaching their peak values at 400 °C. Among all groups, the group with 0.3% PPF exhibits the optimal performance at 400 °C. At this temperature, the hydration reaction inside the matrix is fully completed under the promotion of high temperature, achieving the maximum matrix strength, with the initial cracking strength reaching 18.88 MPa and the peak strength reaching 38.82 MPa. When the temperature exceeds 400 °C, high temperature causes deep dehydration and decomposition of hydration products of cement paste, and cracks form inside the highly dense matrix due to internal steam pressure, which leads to strength reduction. Meanwhile, HF gradually becomes brittle with the increase in temperature, and its bridging and crack-resisting effect keeps weakening, ultimately resulting in a continuous decline in both initial cracking strength and peak strength [42].

In the test, the flexural toughness of each group exhibited a variation pattern different from that of strength. For PPF contents ranging from 0.2% to 0.4%, flexural toughness continuously decreased as the temperature increased from 20 to 800 °C [43]. At the same temperature, flexural toughness also decreased gradually with increasing PPF content. Although the temperature range of 20 °C to 400 °C can enhance strength, high temperature has already caused slight internal damage to the matrix. The pore channels left by melted PPF provide potential paths for subsequent crack propagation, resulting in the failure of toughness to rise synchronously with strength. Above 400 °C, the accelerated propagation of microcracks, interface failure, and HF embrittlement caused by high-temperature damage reduce the energy dissipation capacity of the material, leading to a sharp drop in toughness. At 800 °C, a large number of internal cracks in the matrix connect with each other, and the material can only maintain basic bearing capacity with a remarkable loss of energy dissipation capacity. During the test, rapid crack development occurred, which brought about a drastic reduction in strength. In addition, the agglomeration caused by the increase in PPF content also exerts a negative impact on toughness. When the PPF content rises from 0.2% to 0.4%, fiber agglomeration becomes increasingly obvious. The internal defects formed by agglomerates serve as preferential crack propagation paths under bending load, which weakens the energy dissipation effect of fiber bridging [44]. Moreover, numerous pore channels formed after high-content fibers melt further weaken the continuity of the matrix, resulting in a continuous decline in toughness with the increase in fiber content at the same temperature. In conclusion, flexural toughness is significantly more sensitive to high-temperature damage and defects caused by fiber dosage than strength. The 0.2% PPF group exhibits relatively superior toughness retention capacity across all temperature gradients, while the 0.3% PPF group boasts remarkable advantages in strength indicators. In practical engineering applications, the appropriate PPF dosage should be selected according to the core requirements of high-temperature design, namely prioritizing strength guarantee or toughness guarantee.

It should be noted that the optimum PPF dosage identified in this study is specific to the fiber system investigated, including the adopted HF geometry, PPF diameter, and fiber contents. Fiber geometry, aspect ratio, and volume fraction can substantially affect tensile performance, crack bridging, and fiber–matrix interaction [45,46]. Therefore, the optimum dosage obtained herein should not be directly generalized to other fiber systems without further experimental verification.

### 3.4. Microstructural Characteristics Analysis

Changes in the mechanical properties of UHPS after exposure to high temperatures depend on the degradation degree of fibers and matrices, and the microstructural damage of both serves as the key internal factor determining the evolution law of mechanical properties. Focusing on these two aspects, this study compares the microscopic morphologies of fibers and matrices at different temperatures. The SEM observations are combined with the macroscopic mechanical results to investigate the microstructural evolution of UHPS under high-temperature exposure and its relationship with the observed mechanical behavior.

#### 3.4.1. Changes in Matrix Under High Temperature

**Figure 11:**
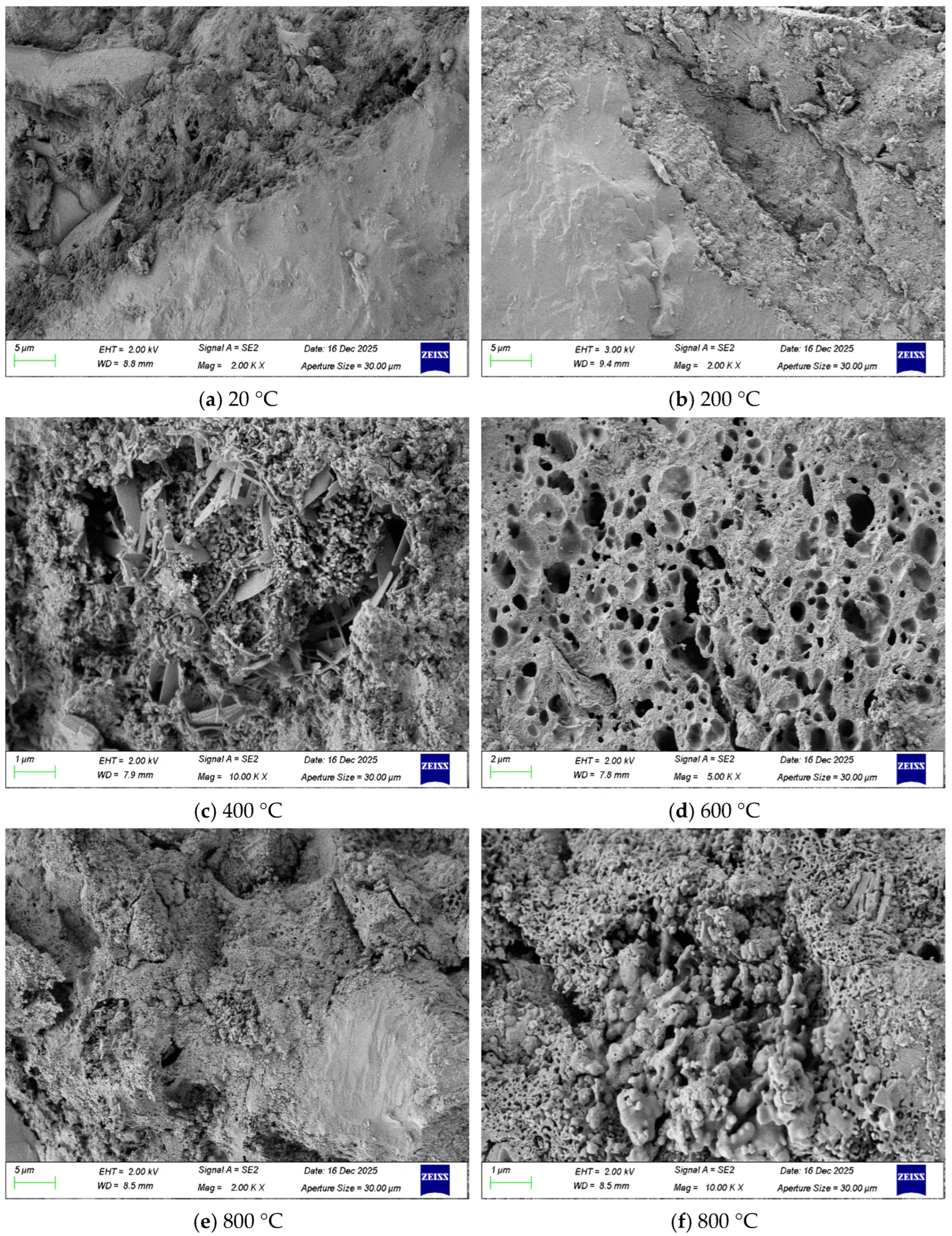
Matrix morphology at different temperatures.

Combined with the SEM observation images of the 0.3% PPF group at different temperatures (20 °C, 200 °C, 400 °C, 600 °C, 800 °C), as shown in Figure 11, at 20 °C, the matrix in the 0.3% PPF group features dense, continuous C-S-H gel and uniformly distributed Ca(OH)_2_ crystals, providing an exceptionally stable micro-foundation for superior mechanical properties.

At 200 °C, the matrix hydration products undergo further hydration. The C-S-H gel maintains a denser flocculent morphology with its three-dimensional network structure remaining intact. The tabular crystal form of Ca(OH)_2_ stays stable without signs of dissolution or fragmentation. At this temperature, free water has mostly evaporated, yet the stability of hydration products remains unaffected. Combined with fine dispersed pore channels formed by the melting of PPF, the matrix retains good integrity, and the corresponding macroscopic mechanical properties are maintained at a relatively high level.

At 400 °C, the hydration products of the matrix exhibit optimized characteristics, and the strength of UHPS reaches its peak. High temperature accelerates the secondary hydration reaction of cementitious materials, generating more fine flocculent C-S-H gel and ettringite (AFt). These newly formed hydration products further fill the tiny internal pores of the matrix, making the original three-dimensional network structure denser than that at 200 °C. Calcium hydroxide (Ca(OH)_2_) crystals remain in a stable state with no obvious signs of decomposition. The optimization of hydration products at this stage enhances the bonding capacity of the cementitious system, and the interfacial transition zone maintains favorable bonding conditions without obvious microcracks. This is consistent with the rule that the macroscopic mechanical properties of the 0.3% PPF group reach the maximum values at 400 °C, including a compressive strength of 135.95 MPa, a splitting tensile strength of 23.24 MPa and a flexural strength of 27.42 MPa.

At 600 °C, the hydration products of the matrix undergo significant decomposition and deterioration, leading to a rapid drop in the strength of UHPS. Typical honeycomb characteristics of the microstructure can be clearly observed in Figure 11d. Flocculent C-S-H gel starts to dehydrate and shrink, changing from dense to loose in morphology, and the original continuous three-dimensional network structure fractures in many places. Plate-like Ca(OH)_2_ crystals begin to decompose, with blurred edges appearing on part of the crystals [47]. The decomposition and shedding of hydration products form a large number of irregular micro-pores inside the matrix. These interconnected pores create a honeycomb-like structure, which greatly reduces the cementing capacity of the cementitious system. Meanwhile, the number of microcracks inside the matrix increases and extends around the pores, and cracks also emerge in the interfacial transition zone due to structural deterioration. The decomposition of hydration products, formation of honeycomb structures, and propagation of microcracks directly contribute to the rapid decline in macroscopic mechanical properties. These changes demonstrate the close relationship between hydration-product degradation, microstructural deterioration, and strength loss.

At 800 °C, the hydration products of the matrix further decompose and fail. As can be observed in Figure 11e,f, the C-S-H gel is completely dehydrated and decomposed, leaving only a small amount of loose amorphous granular products, and the original three-dimensional network structure is thoroughly destroyed. Calcium hydroxide crystals have fully decomposed and disappeared, with no original plate-like morphology observed. The complete failure of hydration products deprives the cementitious system of its bonding capacity, forming a large number of interconnected cracks and pores inside the matrix. The cracks expand remarkably in size and connect with one another, leading to the total failure of the interfacial transition zone. Such severe decomposition of hydration products and microstructural damage result in a sharp decline in macroscopic mechanical properties, including a substantial drop in compressive strength and nearly complete loss of toughness. This reveals the essential reason for the drastic reduction in the bearing capacity of UHPS materials under the high temperature of 800 °C from a microscopic perspective.

#### 3.4.2. Changes in PPF Under High Temperature

**Figure 12:**
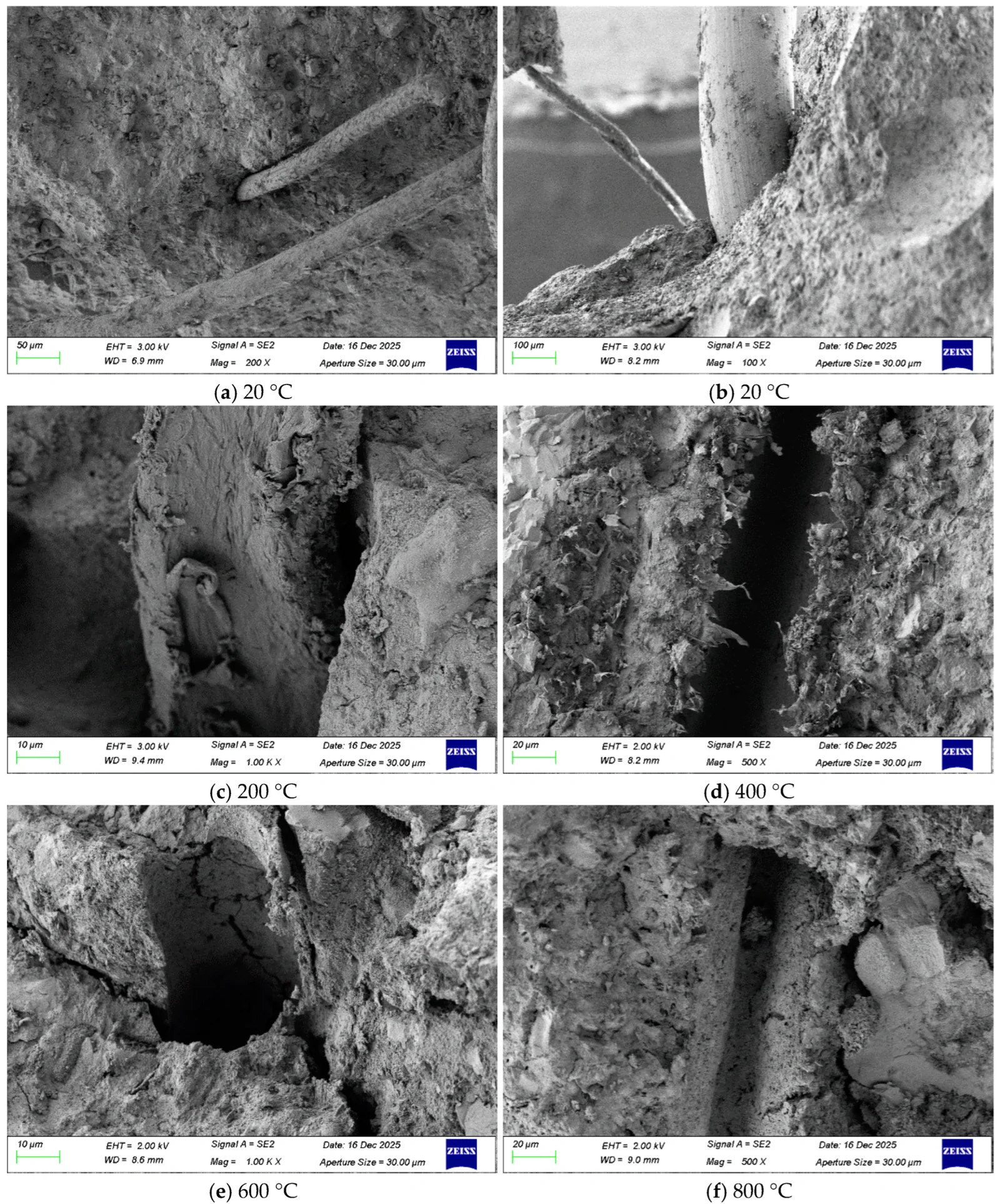
Changes in PPF during high temperature.

As illustrated in Figure 12a, at a normal temperature, PPF is observed within the matrix as intact linear filaments characterized by smooth, defect-free surfaces. These fibers exhibit robust bonding with the surrounding cementitious matrix, displaying no evident interfacial voids. Furthermore, Figure 12b reveals an interwoven distribution of HF and PPF. HF penetrates the matrix, establishing a rigid skeletal structure, while the flexible linear PPF interweaves with them. This synergistic effect constructs a three-dimensional reinforced network, thereby enhancing the mechanical performance of the matrix [48]. The interwoven configuration of HF and PPF serves as a primary microscopic mechanism contributing to the superior mechanical properties observed in the 0.3% PPF group at normal temperature.

At 200 °C, PPF undergoes an initial phase of melting but remains only partially liquefied. As shown in Figure 12c, gaps emerge at the fiber–matrix interface, and PPF generates dispersed circular channels. Concurrently, the nascent melt-induced channels assist in the dissipation of internal vapor pressure, thereby preserving the stability of the hydration products. This aligns with the observation that the mechanical strength remains stable at 200 °C.

At 400 °C, the PPF is completely melted, although some molten residues persist within the matrix. Figure 12d shows no residual intact PPF, with the microstructure characterized primarily by uniformly distributed circular or elliptical molten channels. Residual flocculent or granular PPF components are observed adhering to the channel walls, maintaining favorable interfacial contact with the cementitious matrix. This configuration facilitates the effective release of high-temperature vapor pressure, thereby mitigating internal pressure-induced microcracking while preserving the load-bearing integrity of the matrix. Furthermore, these molten residues appear not to adversely affect the stability of hydration products, such as C-S-H gel and Ca(OH)_2_. Consequently, the specimens exhibit optimal mechanical properties at 400 °C.

Between 600 and 800 °C, PPF is fully melted and most molten residues are evacuated. The resulting channels progressively deteriorate as the temperature increases. As evidenced in Figure 12e,f, the diameter of these molten channels expands significantly at 600 °C, with localized interconnection occurring. The channel walls appear cleaner yet increasingly irregular, likely due to the decomposition of matrix hydration products. By 800 °C, these molten channels coalesce with internal matrix cracks and pores to form a large-scale interconnected network, rendering the original geometry of the channels unrecognizable [49]. During this stage, the transition from partial connectivity to total penetration of the channels disrupts the continuity of the matrix microstructure, facilitating the propagation of internal cracks. Coupled with the thermal deterioration of the matrix—specifically the formation of a honeycomb structure at 600 °C and the complete decomposition of hydration products at 800 °C—the macroscopic mechanical properties shift from a gradual decline to severe attenuation. The strength of the 0.3% PPF group diminishes by 21% at 600 °C and degrades to minimal residual strength at 800 °C, confirming the substantial loss of load-bearing capacity under advanced high-temperature exposure.

#### 3.4.3. Changes in HF Under High Temperature

**Figure 13:**
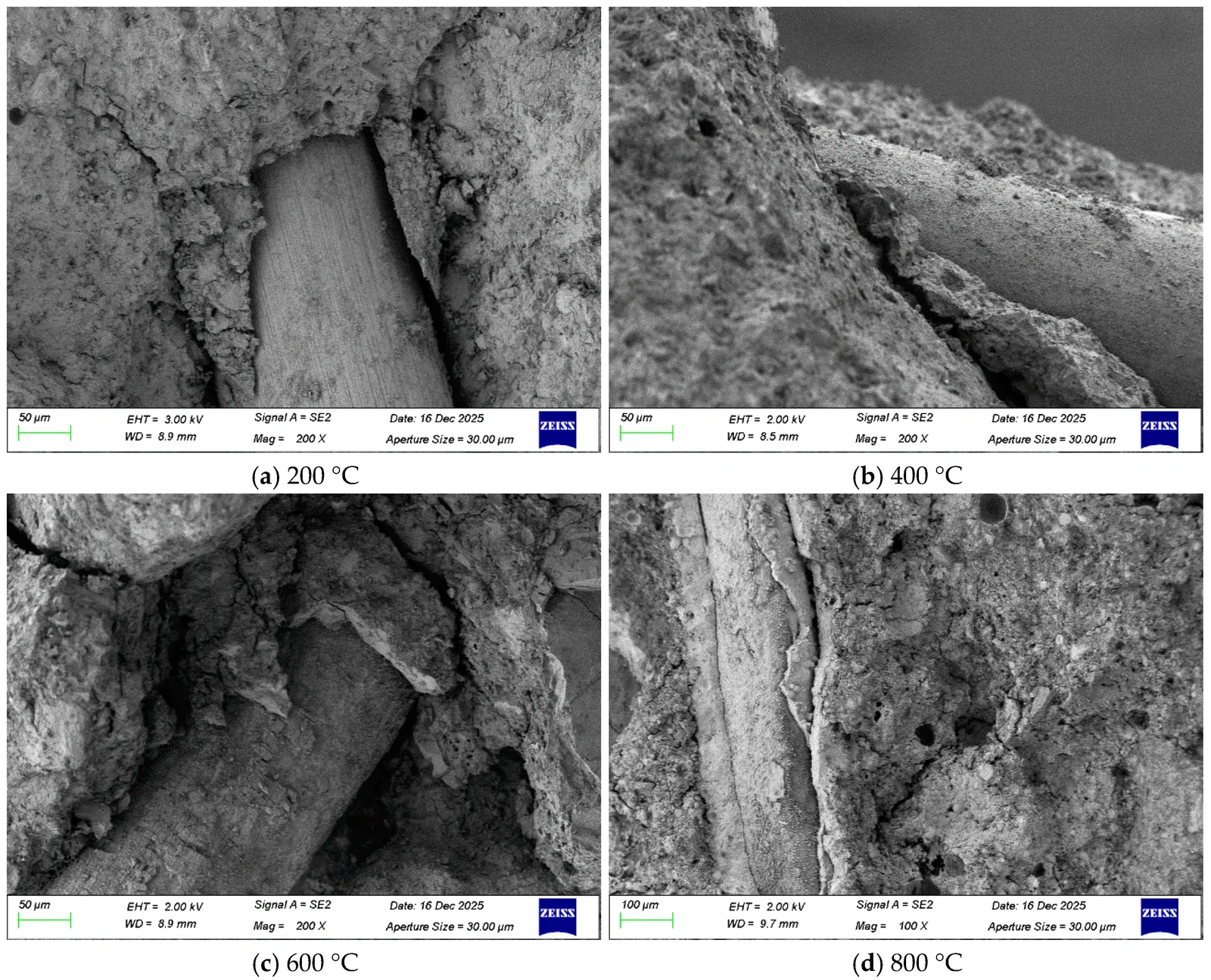
Changes in HF during high temperature.

Based on the SEM images of steel fibers across the 200 °C, 400 °C, 600 °C, and 800 °C temperature gradients presented in Figure 13, the morphological evolution of steel fibers and the gradual failure of their interfacial bonding with the matrix are clearly evident. These microscopic transitions are closely correlated with the attenuation observed in macroscopic mechanical properties [50].

At 200 °C, steel fibers retain a uniform linear morphology with clean, smooth surfaces, devoid of oxidation or corrosion. A robust interfacial bond with the cementitious matrix is maintained, with no visible gaps. During this stage, steel fibers remain thermally stable and operate synergistically with the partially melted PPF to provide effective bridging and restraint. Together with the stable hydration products and the pressure-relief channels formed by PPF, the matrix integrity is well-preserved, which serves as a microscopic basis for the sustained macroscopic mechanical performance at this temperature.

At 400 °C, the morphology of steel fibers in Figure 13b remains largely intact, exhibiting only marginal surface oxidation and the presence of slight light-gray deposits, with no evidence of significant corrosion or cross-sectional degradation. The interfacial bond remains efficient, showing no signs of debonding. Despite minimal oxidation, the stiffness and anchoring capabilities of the steel fibers persist, providing essential reinforcement that supports the attainment of peak mechanical properties at 400 °C.

At 600 °C, the high-temperature degradation of steel fibers, shown in Figure 13c, becomes more pronounced, characterized by the formation of a continuous dark-gray oxide layer and localized spalling. The interfacial bond between the steel fibers and the matrix exhibits significant deterioration, with visible interfacial gaps and incipient delamination. Simultaneously, the decomposition of matrix hydration products leads to the development of a honeycomb structure and widespread microcracking, which further compromises the fiber–matrix bonding efficiency. Moreover, the spalling of the oxide layer reduces the effective cross-section and bridging efficiency of the steel fibers, directly contributing to the rapid decline in macroscopic mechanical properties observed at 600 °C.

At 800 °C, the steel fibers undergo severe thermal degradation, as depicted in Figure 13d. The thickness of the surface oxide layer increases substantially, accompanied by extensive corrosion. Certain steel fibers exhibit cross-sectional thinning and local fracture. The interfacial bonding with the matrix is essentially destroyed, resulting in wide interfacial cracks; the steel fibers become effectively detached from the matrix. Furthermore, the complete decomposition of hydration products creates a large-scale interconnected pore network that no longer provides adequate confinement for the fibers. The synergy between the loss of intrinsic fiber strength, reduced bridging capacity, and the destruction of the matrix microstructure results in a sharp reduction in mechanical performance, leaving only negligible residual load-bearing capacity [51]. This microscopic analysis provides critical insight into the mechanical failure mechanisms of these materials under extreme high-temperature conditions.

## 4. Conclusions

This study investigated the performance of UHPS across four PPF dosages under various high-temperature gradients. The research explored the evolution of appearance, mass loss, and mechanical properties at elevated temperatures, elucidating the functional mechanisms of PPF in UHPS to support the design of high-temperature-resistant materials. The primary findings are summarized as follows:

(1) As temperatures increase, the specimen color shifts from gray to off-white, reflecting internal moisture loss and hydration product changes. The spalling and cracking of UHPS are related to temperature and PPF. No bursting was observed at or below 400 °C, whereas specimens without PPF experienced spalling at 400 °C. Severe bursting occurred in 0% PPF groups at 600–800 °C, while the incorporation of PPF prevented such damage through pressure-relief channels. Mass loss increased with temperature and fiber dosage, peaking at 13.3% for the 0.4% PPF group at 800 °C, indicating that PPF preserves structural integrity at the cost of melting-induced mass loss.

(2) At normal temperature, both compressive and splitting tensile strengths initially increase and then decrease with rising PPF content, reaching peak values of 128.73 MPa and 20.04 MPa, respectively, at a 0.3% dosage. Flexural strength also shows a slight improvement at 0.3% PPF content, achieving 23.86 MPa. The 0.2% PPF group exhibits the highest flexural toughness, suggesting that optimal fiber content varies depending on the specific mechanical property under evaluation.

(3) High-temperature mechanical properties are highly sensitive to thermal exposure and fiber content. Compressive, splitting tensile, and flexural strengths demonstrate an initial increase followed by a decrease, peaking at 400 °C (with values of 135.95 MPa, 23.24 MPa, and 27.42 MPa, respectively, for the 0.3% PPF group). Beyond 400 °C, strength declines significantly due to hydration product decomposition. The 0.3% PPF group maintains the highest strength retention at high temperatures. Conversely, flexural toughness decreases continuously with rising temperature; at any given temperature, toughness declines as PPF content increases, with the 0.2% PPF group demonstrating superior toughness retention.

(4) This study elucidated the multi-scale microscopic mechanisms of UHPS through SEM characterization. While aggregate and interfacial structures influence compressive performance, steel fiber morphology significantly regulates tensile and flexural behavior. Matrix hydration products undergo gradient deterioration: secondary hydration at moderate temperatures enhances density, whereas high-temperature decomposition leads to increased porosity and structural degradation. PPF melts progressively to form multi-scale pressure-relief channels, while steel fibers experience a transition from slight oxidation to severe corrosion and interfacial debonding. The synchronized evolution of these microscopic features and interfacial bonding states governs the macroscopic mechanical behavior of hybrid fiber-modified UHPS under high-temperature conditions.

## 5. Limitations and Future Work

Despite the comprehensive investigation of UHPS in terms of fresh properties, mechanical performance, high-temperature behavior, and microstructural evolution, several limitations remain. First, the thermal properties of fibers and the pore structure of UHPS were not directly characterized. Future work will evaluate fiber thermal stability and conductivity and quantitatively analyze pore structure and porosity to clarify their influence on high-temperature behavior and residual strength.

Second, although SSFA was pretreated by means of natural aging and grinding, the effects of MgO and free-CaO on long-term volume stability remain unclear. Future studies will conduct soundness and expansion tests to assess potential delayed expansion of UHPS with high SSFA content, especially under thermal exposure and service conditions.

Finally, the durability of fiber-reinforced UHPS was not addressed. Future research will examine PPF- and HF-reinforced UHPS under freeze–thaw cycles, chloride attack, wet–dry cycles, and long-term thermal exposure to evaluate its long-term engineering performance.

## Figures and Tables

**Table 1:** Chemical composition and physical properties of cement.

Project	Detection Indicators	Standard Value	Measured Value
Physical properties	Initial setting time (min)	≥45	168
	Final setting time (min)	≤600	229
	Loss on ignition (%)	≤5.0	1.55
	3 d compressive strength (MPa)	≥22	30.7
	28 d compressive strength (MPa)	≥52.5	59.3
	3 d flexural strength (MPa)	≥4.5	5.9
	28 d flexural strength (MPa)	≥7.0	8.8
Chemical composition (%)	MgO	≤5.0	1.01
	SO_3_	≤3.5	2.24
	Cl^−^	≤0.06	0.018
	Clinker + Gypsum	80–94	90.93
	Grinding aid	≤0.5	0.1

**Table 2:** Chemical composition and physical properties of mineral admixtures.

Project	Detection Indicators	Silica Fume	Fly Ash	Metakaolin
Chemical composition (%)	SiO_2_	96.6	50.84	54.28
	Al_2_O_3_	-	33.50	43.83
	Na_2_O	-	0.3	-
	K_2_O	-	0.9	-
	Cl^−^	0.125	-	-
	SO_3_	0.1	0.1	-
	f-CaO	-	0.68	-
Physical properties (%)	Activity Index (7 d)	-	-	117
	Activity Index (28 d)	-	-	123
	Whiteness	-	-	82.2
	Moisture	1.20	0.11	0.25
	Loss on ignition	1.20	0.77	-
	Water demand ratio	109	99	-
	Fineness	-	14.2	-

**Table 3:** Main chemical composition of SSFA (mass %).

Ingredients	Fe	CaO	SiO_2_	MgO	Al_2_O_3_	P_2_O_5_	ZrO_2_
Content	24.5	48.5	12.1	6.9	4.5	1.55	0.79

**Table 4:** PPF Physical Properties.

Project	Tensile Strength (MPa)	Elastic Modulus (GPa)	Elongation at Break (%)	Length (mm)	Diameter (mm)	Melting Point (°C)
PPF	800	5.17	38	12	0.02	160
HF	2950	200	-	13	0.2	-

**Table 5:** Mixing ratio design (kg/m^3^).

Group	Cement	Silica Fume	Fly Ash	Metakaolin	MS	SSFA	HF	PPF	Water
0% PPF	840	120	120	120	270	810	156	0	240
0.2% PPF	840	120	120	120	270	810	156	1.82	240
0.3% PPF	840	120	120	120	270	810	156	2.73	240
0.4% PPF	840	120	120	120	270	810	156	3.64	240

Note: 0% PPF means the PPF content is 0%, 0.2% PPF means the PPF content is 0.2%, and so on.

**Table 6:** Bending toughness parameters.

Fiber Content	Temperature (℃)	Initial Cracking Deflection (mm)	Initial Cracking Strength (MPa)	Peak Deflection (mm)	Peak Intensity (MPa)	Ω_δ_	Ω_3δ_	I_5_
0%	20	1.06	18.24	1.72	23.81	32.90	173.36	5.27
	200	0.68	10.43	1.6	17.82	11.23	79.82	7.11
	400	1.08	16.04	1.34	18.26	29.12	124.97	4.29
	600	—	—	—	—	—	—	—
	800	—	—	—	—	—	—	—
0.2%	20	0.66	10.40	1.62	18.11	11.19	80.34	7.18
	200	0.73	11.33	1.68	19.69	13.83	94.61	6.84
	400	1.07	16.42	2.35	24.84	28.99	174.10	6.01
	600	0.96	10.87	1.85	15.33	17.24	94.04	5.45
	800	0.91	10.18	1.25	11.35	14.86	35.82	2.41
0.3%	20	1.12	15.56	2.33	23.86	25.50	165.62	6.50
	200	1.01	16.66	1.96	24.58	27.92	161.71	5.79
	400	1.25	18.88	2.29	27.42	38.82	201.81	5.20
	600	1.18	15.47	1.94	19.85	30.68	137.12	4.47
	800	0.98	11.86	1.37	13.07	18.46	42.76	2.32
0.4%	20	0.71	10.87	1.33	15.69	12.60	72.84	5.78
	200	1.27	11.64	1.97	16.52	23.42	126.34	5.39
	400	1.16	14.43	1.93	17.70	26.84	118.16	4.40
	600	0.87	10.73	1.15	12.76	18.14	70.99	3.91
	800	0.8	9.71	0.91	9.95	12.94	30.12	2.33

## Data Availability

The original contributions presented in this study are included in the article. Further inquiries can be directed to the corresponding author.
